# Fabrication of Thin-Wall Structures with a Femtosecond Laser and Stainless Steel Powder †

**DOI:** 10.3390/mi15040444

**Published:** 2024-03-26

**Authors:** Iñigo Ramon-Conde, Luis Omeñaca, Mikel Gomez-Aranzadi, Enrique Castaño, Ainara Rodriguez, Santiago M. Olaizola

**Affiliations:** 1 CEIT-Basque Research and Technology Alliance (BRTA), Manuel Lardizabal, 15, 20018 Donostia/San Sebastián, Spain; 2 Departamento de Ingeniería Eléctrica y Electrónica, Universidad de Navarra, Tecnun, Manuel Lardizabal, 13, 20018 Donostia/San Sebastián, Spain

**Keywords:** additive manufacturing (AM), thin profiles, femtosecond laser, precision manufacturing, micron-scale features

## Abstract

Additive Manufacturing (AM) has revolutionized the production of complex three-dimensional (3D) structures; however, the efficient and precise fabrication of thin profiles remains a challenge. This study explores the application of femtosecond-laser-based additive manufacturing techniques for the production of thin profiles with micron-scale features, reaching profile thicknesses below 100 µm. The study investigates the effects of scanning strategy, with optimized processing parameters, on the fabrication of thin profiles; wall thickness measurements were carried out using various technologies to analyse the influence of each on the resulting values. The quality of the walls was quantified by means of a visual characterization of the melted volumes, analysing the evolution of the measured thickness with regard to the processing conditions and in relation to the theoretical thicknesses of the walls.

## 1. Introduction

In recent years, the use of Additive Manufacturing (AM) has become part of the daily life of a wide range of industries [1]. As the demand for more precise, intricate, and superior parts continues to rise across diverse applications and sectors, the evolution of AM research is expanding the scope of fabrication of a broader range of components.

One of the areas of research within AM that is growing faster than others is micro-scale Additive Manufacturing (mAM) [2]. While micro-mechanisms can be developed, the technologies mastered for manufacturing parts on a macro scale face the challenge of reducing the size of the products, as the need for tiny metal parts increases. Multiple industrial sectors—not only classical ones like micro-mechanical or micro-fluidic sectors, but also the medical industry for implants—demand a higher technological level and smaller-sized parts to face future challenges and advance in the development of new technologies [3]. However, there are still significant challenges that exist in terms of available materials, resolution, throughput, and ability to fabricate true three-dimensional geometries [4].

The first drawback that must be overcome in micro-technology is scaling. Depending on the process type, the scale dimensions may vary, with each scale imposing specific standards for surface quality and dimensional accuracy. Traditional scaling poses significant difficulties, as conventional technologies often operate beyond their designated “design zone”, necessitating structural modifications to achieve size reduction in the final parts. In the present work, we explore alternative methods to established AM technologies due to their limitations in terms of downscaling [5].

The disadvantages of using well-established AM technologies, though, bring the opportunity to consider the use of other unconventional methods; the introduction of ultrashort pulse (UPS) lasers brings more precision and enables us to control more precisely the amount of heat submitted to the material. As demonstrated in a previous study [6], the accumulation of significant amounts of heat onto a stainless steel powder substrate using a femtosecond (UPS) laser is possible, yet does not have the melting capacity of continuous wave or long-pulsed lasers. Despite this, and compared to them, USP lasers bring more precise heat accumulation control, leading to a more flexible melt pool; thus, higher spatial resolution is achieved, permitting the fabrication of thinner and more complex structures [7]. Moreover, additive manufacturing studies with femtosecond lasers are small [8], and even smaller with stainless steel powder, so this study analyses a field still unexplored.

This study delves into the potential of using a femtosecond laser as a tool for crafting thin-walled structures, continuing the work presented previously in [9] and providing a more extensive analysis of the morphology of the structures and methods of wall thickness analysis. Femtosecond lasers offer notable advantages in the precise control of heat accumulation within the material, resulting in a more adaptable melt pool. Consequently, this enhances spatial resolution, opening up possibilities for manufacturing thinner and more intricate structures, reaching walls of less than 100 µm, which can be achieved with a shorter pulse duration than in other studies [10,11,12]. The present investigation aims to explore the efficacy of femtosecond lasers in pushing the boundaries of micro-scale additive manufacturing, addressing key challenges and contributing to the evolution of advanced manufacturing technologies.

## 2. Materials and Methods

Self-produced gas atomized AISI 316L stainless steel powder [13] grains were used, with a size distribution up to 20 µm; the use of this powder is an advantage over commercial formulations, as it allows the selection of a suitable size and morphology to facilitate and improve the melting process and obtain more effective results. AISI 304L stainless steel cut into slabs of 125 × 100 × 5 mm was selected as a substrate on which to deposit the powder and carry out the processes.

To obtain the optimal conditions inside the melting regime, a simulation based on the Two-Temperature Model was conducted [14]. This model postulates the existence of two distinct temperatures in the ultrafast laser–material interaction.

(1) $$C_{e}\frac{\partial T_{e}}{\partial t}=\nabla \left[\kappa_{e}\nabla \left(T_{e}\right)\right]-G\left(T_{e}-T_{l}\right)+Q\left(r,t\right),$$

(2) $$C_{l}\frac{\partial T_{l}}{\partial t}=\nabla \left[\kappa_{l}\nabla \left(T_{l}\right)\right]+G\left(T_{e}-T_{l}\right)$$

The subscripts *e* and *l* refer to the electron and lattice parameters, respectively; C is the heat capacity, T is the temperature, κ is the conductivity and G is the electron–lattice coupling factor. Based on the model proposed by Zhang [15], $C_{e}=C_{e}^{\prime }T_{e}$ and $C_{e}^{\prime },C_{l},\kappa_{e},\kappa_{i}$ and G can be considered constants with temperature. The energy absorption rate Q can be decomposed into a spatial and a temporal component:

(3) $$Q\left(r,t\right)=S\left(r\right)T(t),$$

where

(4) $$S=\frac{1-R}{\delta +\delta_{b}}F{\left(\frac{w_{0}}{w}\right)}^{2}\exp\left(\frac{z-z_{s}}{\delta +\delta_{b}}-2\left(\frac{({r-r_{0})}^{2}}{w^{2}}\right)\right),$$

(5) $$T=\frac{1}{t_{p}}\sqrt{\frac{4Ln\left(2\right)}{\pi }}\exp\left(-4Ln\left(2\right){\left(\frac{t-2t_{p}}{tp}\right)}^{2}\right).$$

Here, *R* is the reflectance of the metal, *δ* is the optical penetration depth; *F* is the laser fluence, $w_{0}$ is the spot size and w takes into account the spatial extent of the laser beam, as indicated in Equations (6) and (7), where $Z_{R}$ is the Rayleight length and $t_{p}$ is the FWHM pulse duration.

(6) $$w\left(z\right)=w_{0}{\left(1+{\left(\frac{z}{Z_{R}}\right)}^{2}\right)}^{1/2},$$

(7) $$Z_{R}=\frac{n\pi w_{0}^{2}}{\lambda }.$$

The finite-difference method was used to solve Equations (1) and (2). The FDM is based on approximating the differential equations using finite differences. In this study, due the lack of symmetry and the coupling between both equations, an explicit method was employed, and this technique allows the calculation of the temperature values for the time step *t* + 1 using the values at the same grid point and the first neighbours at the time *t*. Due to rotation symmetry of laser ablation, a 2D simulation model was developed to simplify the simulation and improve the computational speed. Every simulation started at time t=0, and initial conditions for both electron and lattice temperatures were fixed at ambient temperature $T_{0}=300\;K$. Given the process timescale, it is plausible to disregard any significant heat losses from the film to its surrounding environment. As a result, the initial boundary conditions can be accurately characterized by

(8) $$T_{e}\left(r,z,t=0\right)=T_{l}\left(r,z,t=0\right)=T_{0},$$

(9) $${\left.\frac{\partial T_{e}}{\partial n}\right|}_{\Omega }={\left.\frac{\partial T_{l}}{\partial n}\right|}_{\Omega }=0$$

where Ω represents the boundary surfaces of the film. Knowing the evolution of both temperatures allows for the precise prediction of the melting and ablation profile in the stainless steel for different laser powers. Stainless steel has a melting temperature of $T_{Melt}=1648\;K$, while ablation happens when the lattice temperature is above $0.9\;T_{c}.\;$ For stainless steel, the critical thermodynamic temperature is $\;T_{c}=10,360\;K$. Ablation due to such a high temperature results in extremely high pressure that will be released through the adiabatic expansion in the ablated region. The simulation was performed using custom code developed in Python.

The irradiation source was a diode-pumped ultra-fast fibre laser system (Amplitude Satsuma HP) of λ = 1030 nm and 280 fs pulse duration, with a maximum average power of 10 W at a repetition rate of 500 kHz. The properties of the laser beam were modified and controlled by the different modules of the micromachining set-up (LASEA LS-Lab). Finally, an F-theta lens focused the laser beam to a 29.9 µm diameter spot on the processing area. 

To fabricate thin-wall structures, well-defined processing parameters are needed to ensure that the metal powder is efficiently melted. We have based this investigation on our previous studies on AM, and therefore the first parameters used to fabricate thin-wall structures were the best processing conditions from [6], where the melting dynamics of stainless steel powder and femtosecond laser were studied. Control of the heat submitted to the material is crucial; therefore, an analysis was performed regarding the efficiency of the absorption of the energy of the laser pulses into the material.

**Figure 1:**
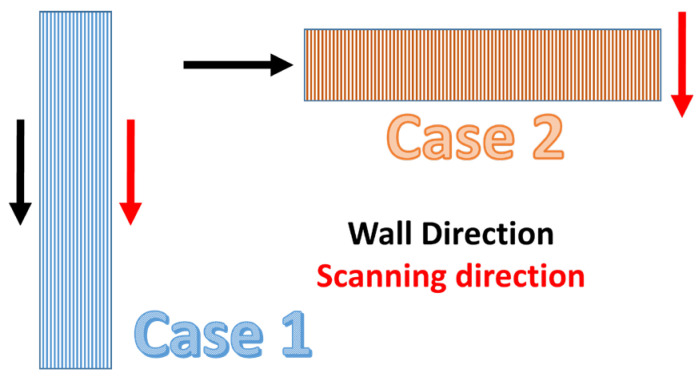
Schematic of the scanning strategies with parallel (Case 1) or perpendicular (Case 2) wall and scanning directions. Arrows in black represent the direction of the wall (either vertical or horizontal); arrows in red represent the scanning direction of the laser.

Two scanning strategies were designed, considering the scanning direction and the wall direction. These strategies were based on two arrangements, as displayed in Figure 1. In the first one, the scanning direction and wall direction were parallel, and therefore the scanning lines had a length of the size (*L*) of the processed wall. In the second one, the scanning direction and wall direction were perpendicular, so scanning lines had the length of the thickness (*t*) of the processed wall. This second strategy forced the laser to process short lines, enabling the scanner to jump rapidly to the next line and enabling the new melted volume to be more easily coalesced with the previous one. 

Using these scanning strategies, a series of different structures was created to study which was the minimum wall thickness that was achievable with the optimized parameters taken from [6], at a pulse repetition rate of 500 kHz. The two sets of parameters used were:

- A—Power: 0.78 W; Scanning Speed: 2.5 mm/s; Hatch Distance: 5 µm.
- B—Power: 0.85 W; Scanning Speed: 2.5 mm/s; Hatch Distance: 7.5 µm.

**Figure 2:**
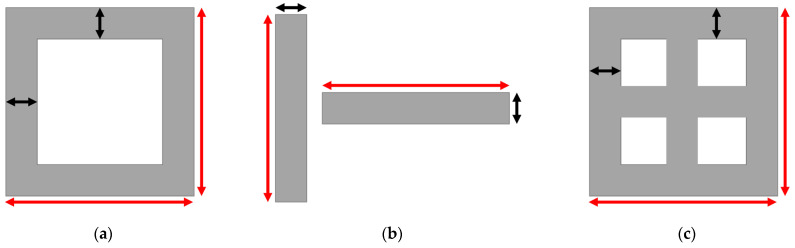
Representation of the empty square (**a**), single wall (**b**) and partitioned square (**c**) designs, where black arrows represent the thickness (*t*) of the walls and red arrows represent the length (*L*) of the walls.

To integrate both scanning strategies in single designs, three layouts were designed: empty squares, single walls and partitioned squares. In all the experiments, the scanning direction was vertical, enabling the aforementioned scanning strategies, depending on the design. In Figure 2, the representation of each design is shown; each type of structure was fabricated with decreasing wall thickness, and all of them used four 50 µm powder layers, resulting in 200 µm height structures. The thickness of the designs ranged from 1 mm to 5 µm with a wall length of 2 mm for empty squares; from 1 mm to 25 µm with a wall length of 4 mm for single walls; and from 50 µm to 5 µm with a wall length of 2 mm for partitioned squares.

The cleaning stage is a major impediment before and during analysis. In the case of small and low-thickness structures, this becomes even more evident given the characteristics of the manufactured profiles. As the profiles are very thin and, hence, their structural strength is notably reduced, cleaning must be carried out carefully. Each cleaning process consisted of three stages, starting from first one and moving on to the next if the degree of cleaning was inadequate:

- Dump the powder on the substrate to allow it to fall with gravity;
- Vacuum up dust;
- Blow away the dust with varying degrees of strength, depending on the cleaning requirement.

Despite the care taken in extracting the powder, many of the samples were damaged; in any case, the cleaning requirements were taken as a strength scale to analyse the properties of the fabricated walls.

Due to the lack of continuity in certain walls, since not all of them had a flat edge that could be precisely quantified, three different techniques were used to evaluate the quality of the processes and to measure the thickness of the profiles: Optical Microscopy (OM) (LEICA M205 FA), Scanning Electron Microscopy (SEM) (ZEISS SIGMA and JEOL JSM 7100F) and non-contact Optical Profilometry (OP) (Sensofar S-Neox). Among the methods used, two groups can be distinguished, depending on the way in which the wall thickness is measured. Firstly, microscopy methods focus on measuring wall thickness from an image of the wall, so although an attempt can be made to obtain the most representative value for each wall, it is always subject to the interpretation of the researcher. Secondly, the optical profilometry method sweeps the wall, creating a topographical map on which different profiles are drawn perpendicular to the wall, and these profiles can extend along the entire length of the wall, obtaining an averaged value of the wall thickness. This makes the second group of measurement techniques possibly the most precise, as they consider the largest percentage of the wall to make a measurement, instead of individual values. Differences between techniques, thus, lie in the interpretation of the images and in the measurement system of the technique.

## 3. Results

The results were divided in two sections; firstly, the results of the simulation will be explained. Secondly, the results of the fabrication of thin-wall structures are detailed.

### 3.1. Two-Temperature Simulation

**Figure 3:**
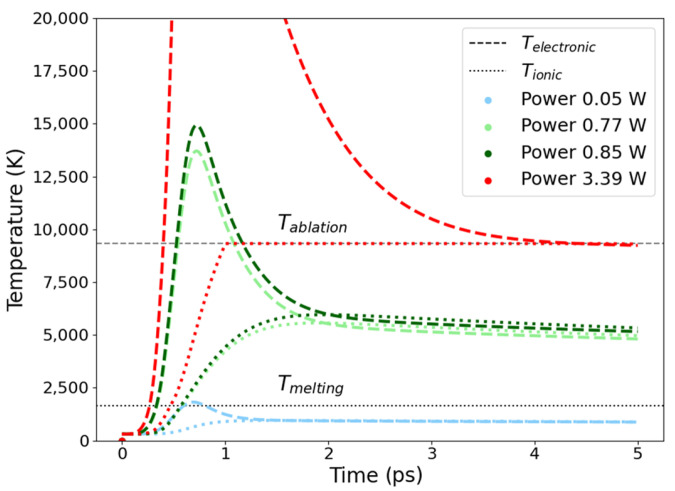
Simulated lattice temperature evolution for different laser powers.

As observed in Figure 3, for the case in which the power is 0.05 W, the lattice temperature does not reach the melting point, resulting in no material melting. On the other hand, for power of 3.39 W, if the temperature surpasses the critical threshold, we will observe ablation. Therefore, choosing the appropriate power level is critical for successful melting.

**Figure 4:**
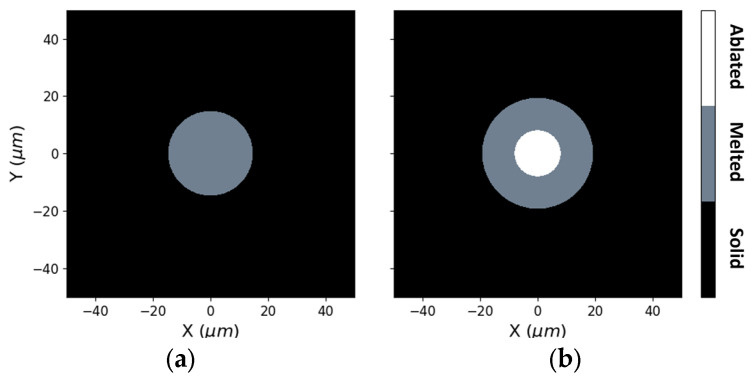
Simulated X-Y profile after the laser pulse with a power of 0.77 W (**a**) and 3.39 W (**b**).

Simulations show that, due to the significantly lower heat incubation capacity of the femtosecond pulses, it is not possible to obtain a melted area broader than each single line. As shown in Figure 4, the melt diameter is nearly equal to the 30 µm spot diameter, indicating that there were no heat diffusion effects at the edges. Nevertheless, this can be an advantage when fabricating thin profiles with high definition. This precise focusing of heat allows a very contained melt zone to be achieved.

### 3.2. Thin-Wall Structure Thickness Analysis

**Figure 5:**
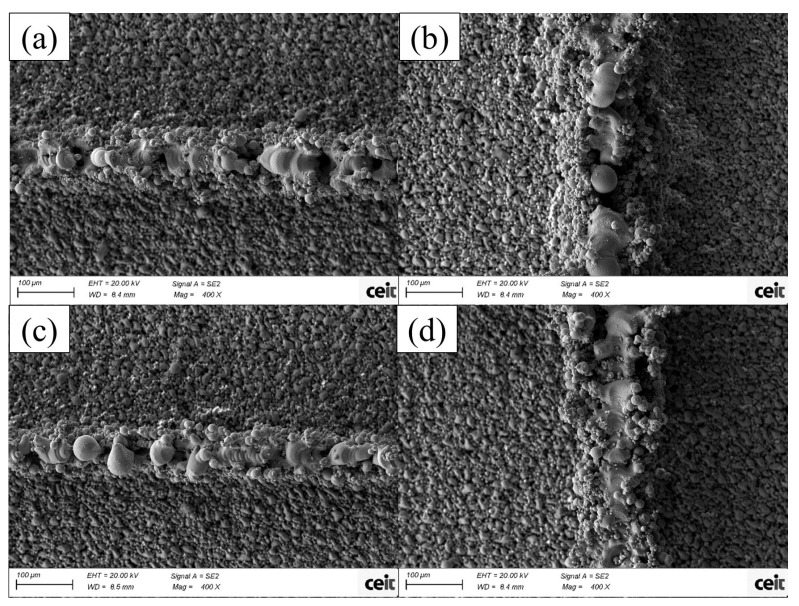
SEM images of empty square walls processed with a femtosecond laser with A-processing conditions. Designs of (**a**,**b**) are 5 µm and designs of (**c**,**d**) are 10 µm of thickness. The resulting thicknesses are (**a**) 110 µm, (**b**) 160 µm, (**c**) 140 µm and (**d**) 200 µm.

A collection of different structures was fabricated with wall thickness values ranging from 5 µm (which corresponded to a single scanning line in some of the cases [16]) to 1 mm, in multiple steps. Figure 5 shows the results of the 5 µm ((a) and (b)) and 10 µm ((c) and (d)) walls from the empty square designs. In the 5 µm wall, the formation of smooth volumes of melted powder are visible with almost no adherence to non-melted particles, although they are not melted in a single continuous structure. In the 10 µm wall, when the scanning directions are perpendicular, the results are similar; in contrast, for the parallel processing, the wall is more uniformly and continuously melted. In both processes the multiple void areas can be explained due to a lack of melted powder in the lower levels, making it impossible to form a stable powder layer when processing successive stages.

**Figure 6:**
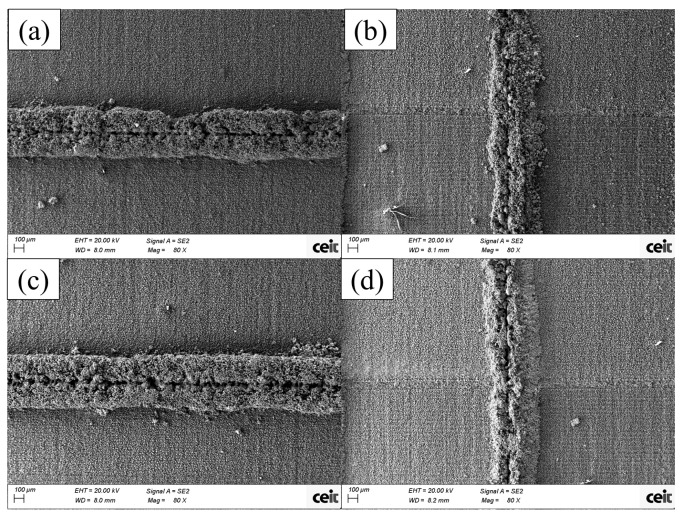
SEM images of single walls processed with a femtosecond laser with B-processing conditions. Designs of (**a**,**b**) are 25 µm and designs of (**c**,**d**) are 50 µm thick. Resulting thickness are (**a**) 500 µm, (**b**) 300 µm, (**c**) 550 µm and (**d**) 240 µm.

In Figure 6, 25 µm and 50 µm thickness single-wall pictures can be seen. For these designs, the structure is formed by a large amount of unmelted powder particles covering the actual melted powder wall; depending on the amount of these leftover metal powders, the wall thickness varies significantly. The internal structure of the wall that lies below the powder particles—easier to appreciate in vertical walls—seems to be formed by independent bubble-like structures in the central line with more continuous borders. Vertically oriented samples are the ones presenting lower thickness values, with around half the thickness value of horizontal walls for the cases presented below; this is mainly due to the leftover amount of powder in horizontal walls. The presence of leftover powder does not allow a clear view of the internal wall structure, as this covers the majority of the wall’s structure; despite this, it is possible to conduct an analysis of the combined thickness of the structure and the powder around it and make a comparison for the different design thickness values.

Finally, it is worth mentioning a phenomenon that occurred in all the samples, at different levels, which is a central line resembling a cut that crossed all the samples in the same direction in each wall, due to pore formation during the fabrication of walls [17]. 

Concerning the partitioned square wall structures, the results are similar to previously analysed designs, but with different powder–wall proportions. In the case of single wall and empty square designs, the walls were formed by an internal melted structure and they were covered by a very thin layer of unmelted powder particles, allowing a relatively clear view of the wall structure; in partitioned square walls, this layer is thicker, preventing a clear view of the shape, size or quality of the internal wall.

**Figure 7:**
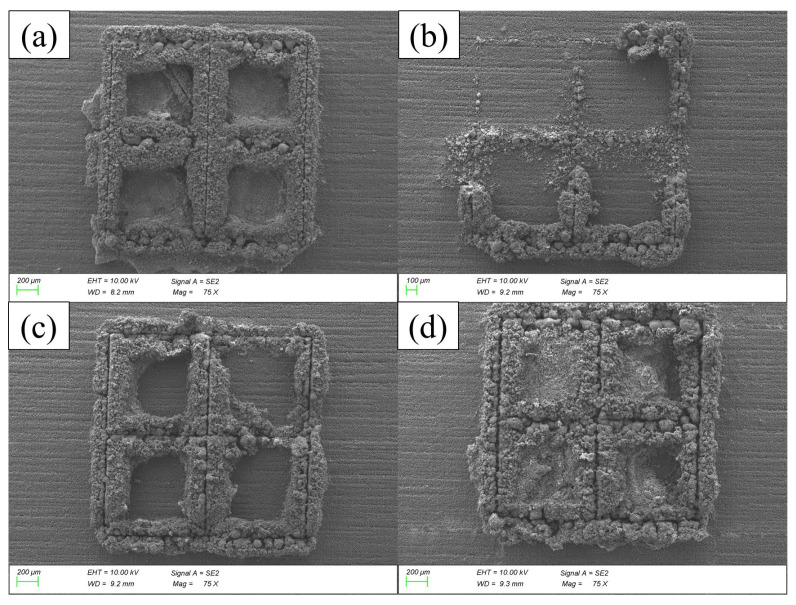
SEM images of single walls processed with a femtosecond laser with A-processing conditions. Designs of (**a**) 5 µm, (**b**) 10 µm, (**c**) 25 µm and (**d**) 50 µm thickness. Resulting average thicknesses are (**a**) 244 µm, (**b**) 305 µm, (**c**) 343 µm and (**d**) 368 µm.

In Figure 7, the set of pictures shows the effect of increasing the wall thickness using the first set of parameters (A). In 5 μm wall thickness structures, there is a lower layer of powder in the base, as observed in Figure 7a, resulting from a very subtle powder cleaning process to try to avoid any damage; when performing a stronger cleaning process, damage can occur, as seen in the top-left corner of Figure 7b. As the wall thickness increases, the strength of the wall increases, becoming less easily damaged during cleaning and permitting stronger powder extraction, which results in clean bases and less powder around the structures; this strengthening is depicted in Figure 7c,d. Finally, with 50 μm walls, the widest and presumably the strongest structures are obtained, which allow the powder to be trapped and make them the least tidy structures among these four.

**Figure 8:**
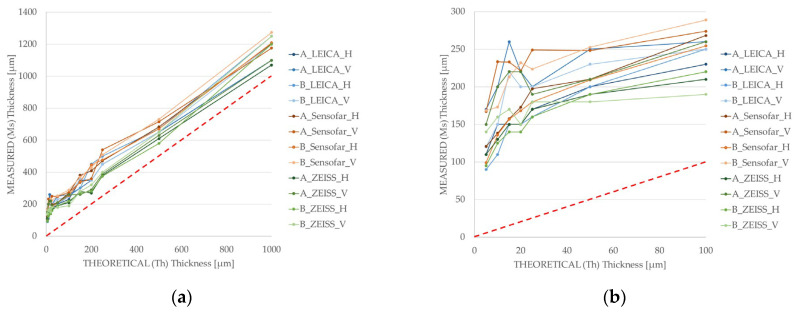
(**a**) Comparison between the theoretical and measured wall thicknesses. (**b**) Augmented view of the comparison between the theoretical and measured wall thicknesses below 100 µm. The red dashed line represents the 1:1 ratio for Ms:Th, for equal value of theoretical and measured wall thicknesses. Measurement techniques used: in blue OM measurements, in orange OP measurements and in green SEM measurements.

Upon analysing the thickness of the fabricated walls, it was concluded that the heat transmitted to the powder when processing singles lines or thin structures was enough to generate melting phenomena. In Figure 8a, the comparison between the theoretical (Th) and measured (Ms) wall thicknesses is shown. As the wall thickness decreases, the Ms:Th ratio increases; for the 1 mm wall thickness the ratio is 1.1–1.25, whereas for the structures below a thickness of 20 µm, the ratio increases up to 10–35. Interestingly, the measured wall thickness is always at least around 90–100 µm higher than the theoretical value (Figure 8b), which can be related to a minimum laser affection area. This increase in the irradiation area is inherent to processing, and occurs in all cases, regardless of the theoretical thickness; the case where it is most clearly seen is in the 5 µm wall, where a single pass of the laser beam generates enough heat accumulation to generate a melt beyond the spot diameter.

In fact, the lowest wall thickness that it was possible to measure was around 90–100 µm, corresponding to the 5 µm wall perpendicular design (Case 2), evidencing that with the current laser setup and beam characteristics, there is a limit on the minimum achievable wall thickness. Interestingly, there is a relatively constant wall thickness value, ranging from 120 to 130 µm to 200 to 220 µm, for design thicknesses of 10, 15, 20, 25, 50 and 100 µm (Figure 8b), where the theoretical wall thickness is increased in small steps, never becoming large enough to affect the heat accumulation area.

## 4. Discussion

The 90–100 µm minimum increase in the size of the theoretical wall thickness value contradicts the results of the simulation, which produced an expected heat-affected zone equal to the laser spot, around 30 µm. It must be noted that the simulations were made assuming a continuous medium instead of a bed of powders, which involved more variables that were not taken into account due to the high complexity they would add to the calculations. The introduction of a discontinuous and randomly ordered medium, such as a powder bed, affects the distribution of the accumulated heat in the process area; supposing the same amount of energy is employed to melt the material in the substrate, a lower-density medium would be melted more broadly in the irradiation area as a consequence of the wider distribution of the same amount of matter (if this was in a solid core). This and the probability of sintering [18] of the metallic powder particles instead of pure melting, are believed to be the main causes for the increase in the heat accumulation area compared to the values obtained in the simulations.

Comparing the different structures, the first conclusion that can be taken is the influence of the design in the final thickness value. The lowest wall thickness values were obtained for empty square designs, which makes sense as these were the group covering a wider range of thickness values. Despite this, single walls present lower values in the 200–1000 μm range; below the 200 μm value and down to 25 μm—the lower limit of single walls—the results tend to converge on the same line. Partitioned square designs, which were only tested in four thickness values, present notably higher measured wall thicknesses, around 200 μm more than empty square designs.

**Figure 9:**
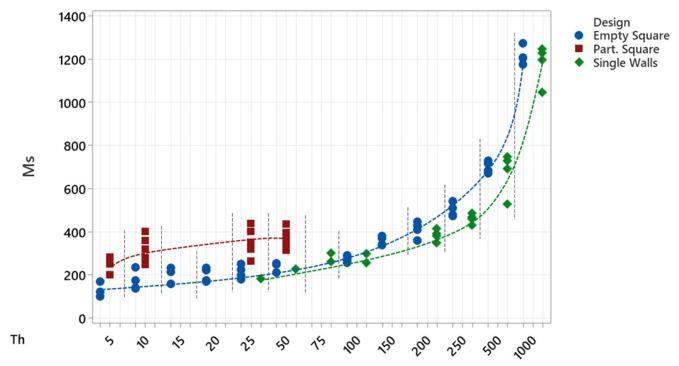
Measured wall thicknesses of single wall (green), empty square (blue) and partitioned square (red) experiments; dotted lines have been drawn to highlight the trend of each structure’s design.

The results in Figure 9 show that, as the design becomes more complex, the thickness of the walls increases; this increase in wall thickness leads to an increase in wall strength and resistance, as could be seen in the cleaning process, where the least damaged structures were the most complex designs. The increase in strength in the walls may enable the possibility of decreasing the wall thickness. Single wall design is the least resistant among the tested structures, as the only support of these structures is their bond to the substrate, making them very sensitive to the cleaning process or other external forces or effects. Partitioned squares are, in contrast, the structures that present the highest strength, but are also the samples with the largest amount of excessive powder particles; their design promotes the accumulation of the powder particles inside the cells, making it difficult to extract the powder out of them and—on some occasions—enforcing an excessive cleaning procedure that causes damage to the lowest-thickness structures.

Finally, empty squares combine the advantages of single wall and partitioned square designs. As the structure is formed by four walls joined at their ends, empty squares have the inter-wall space of single wall designs, but the increased strength of partitioned squares. Furthermore, due to the larger interior space within them compared to the partitioned squares, the extraction of powder is facilitated, resulting in more efficient and thorough cleaning.

**Figure 10:**
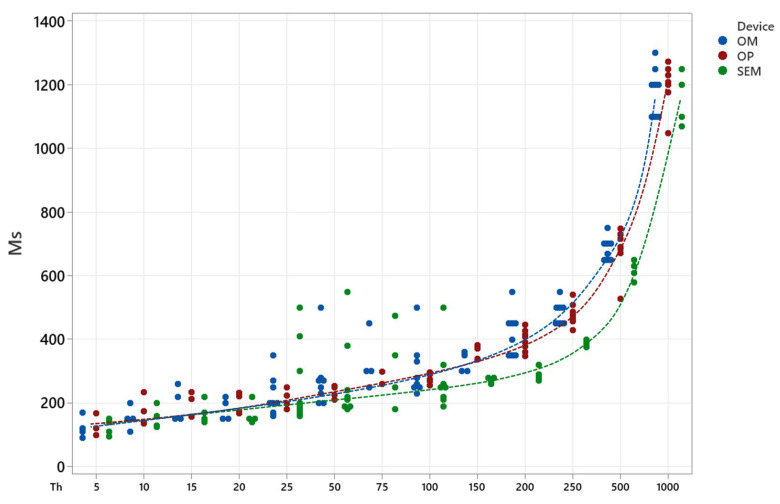
Wall thickness measured in single walls and empty squares using the three different techniques: Optical Microscopy (blue), Optical Profilometry (red) and Scanning Electron Microscopy (green).

Differences between measuring techniques lie in the interpretation of the images and in the measurement systems of each technique. Analysing the independent values together (shown in Figure 10), it can be observed that all of them follow a similar tendency with different degrees of stability on their values. SEM measurements provide the lowest thickness values out of the three techniques; conversely, optical microscope measurements (OM) return the highest values recorded. Both microscopy techniques present great variability in central (25–50–75 μm) values of wall thickness, which are the results of the measurements in a range of 400 μm in thickness, more than four times the original thickness value. Optical profilometry (OP) has the smallest deviation along the whole set of measurements, with some exceptions for satellite values in the largest structures. Moreover, and as a result of the averaging of the data collected in the topographic map of each wall, OP thickness values are located between the values obtained with both microscopy techniques, above SEM and below OM data; due to this, optical profilometry data are understood to be the most reliable.

## 5. Conclusions

In order to study the potential of ultra-short pulse lasers for the fabrication of thin-wall structures, a femtosecond laser was used as an energy source in Laser Powder Bed Fusion (LPBF) processes, in different studies performed with self-produced stainless steel powder particles with a size distribution lower than 20 µm. The conclusions obtained are as follows:

- After performing simulations with the set of reference parameters, it was found that femtosecond laser pulses are able to generate a small melt pool of the same size of the laser spot, indicating that there are no heat diffusion effects at the edges (considering a stainless-steel solid core).
- This study continues the work carried out in [6] and extends the information presented in [9], demonstrating that the femtosecond laser is not only valid for melting metal powder, but also for making complex profiles, and thanks to its controlled heat input, enables very thin profiles to be made, as thin or thinner than other similar techniques. Further research in this topic with higher pulse repetition may offer potential improvements in mAM using LPBF, a technique with a high maturity index.
- A scanning strategy has been found to be decisive in fabricating profiles with reduced thickness. For walls larger than 100 µm, where the thickness of the wall is much larger than the spot diameter, the scanning direction is not important, as the processing parameters are more influential; in contrast, when the wall is not much larger than the diameter spot, different scanning strategies strongly influence the results.
- Structure design is decisive with regard to the structural resistance to the cleaning process, a necessary step in all LPBF processes; the distribution of the walls in the structure also determines the amount of unmelted powder adhering to the structure, which in some cases hampers the measurements of the real wall thicknesses.
- The lowest measured wall thickness in the experiments was 90 µm, for a wall design of 5 µm; the rest of the measured thicknesses were at least around 90–100 µm higher that their theoretical value, denoting significant heat accumulation rates able to generate melting and/or sintering in an area wider than the laser spot.

Finally, it has also been verified that this heat accumulation is higher than expected in the simulations, around three times the calculated value, which is probably due to the differences in the behaviour of the laser radiation between a solid substrate and a powder bed, which indicate broader heat diffusion between powder particles, compared to the solid simulated metal core.

## Data Availability

The raw data supporting the conclusions of this article will be made available by the authors on request.
